# Biomechanical insights into ankle instability: a finite element analysis of posterior malleolus fractures

**DOI:** 10.1186/s13018-023-04432-x

**Published:** 2023-12-12

**Authors:** Jichong Ying, Jianlei Liu, Hua Wang, Yunqiang Zhuang, Tianming Yu, Shuaiyi Wang, Dichao Huang

**Affiliations:** 1 Department of Orthopaedic Trauma, Ningbo No.6 Hospital, Ningbo, China; 2 Department of Medical Imaging, Ningbo No.6 Hospital, Ningbo, China

**Keywords:** Finite element analysis, Posterior malleolus fractures, Stress distribution, Sagittal angle, Ankle instability, Traumatic arthritis, Ankle joint

## Abstract

**Background:**

Posterior malleolus fractures are known to be associated with ankle instability. The complexities involved in obtaining precise laboratory-based spatial pressure measurements of the ankle highlight the significance of exploring the biomechanical implications of these fractures.

**Methods:**

Finite element analysis was utilized to examine the stress distribution across the contact surface of the ankle joint, both in its natural state and under varied sagittal fracture line angles. The study aimed to identify stress concentration zones and understand the influence of sagittal angles on stress distribution.

**Results:**

Three distinct stress concentration zones were identified on the ankle's contact surface: the anterolateral tibia, the anteromedial tibia, and the fracture line. The most significant stress was observed at the fracture line when a fracture occurs. Stress at the fracture line notably spikes as the sagittal angle decreases, which can potentially compromise ankle stability. Larger sagittal angles exhibited only minor stress variations at the contact surface's three vertices. It was inferred that sagittal angles below 60° might pose risks to ankle stability.

**Conclusions:**

The research underscores the potential implications of fractures on the stress profile of the ankle joint, emphasizing the role of the contact surface in ensuring stability. The identification of three zones of stress concentration and the influence of sagittal angles on stress distribution offers a valuable reference for therapeutic decision-making. Further, the study reinforces the importance of evaluating sagittal fracture angles, suggesting that angles below 60° may compromise ankle stability.

## Background

### Introduction and background

Ankle fractures are among the most common musculoskeletal injuries encountered in orthopedic and trauma settings. Representing a significant proportion of injuries, current epidemiological data estimate their involvement in 4%-10% of all orthopedic presentations [1, 2]. Delving deeper, fractures of the posterior malleolus emerge as particularly noteworthy, with a reported prevalence spanning from 10% to a staggering 44% of all ankle fractures [3, 4]. Such statistics become especially compelling when we consider the clinical ramifications associated with these injuries.

The aftermath of posterior malleolar fractures often extends beyond mere skeletal disruption. Their clinical gravity is heightened by the frequent development of complications like traumatic arthritis. This adverse sequela is primarily attributed to the disrupted and intermittent contact of the articular surface, leading to degenerative changes over time [5, 6]. Such clinical observations have not only emphasized the acute intervention, these fractures require but have also accentuated the need to elucidate the underlying pathological mechanisms and biomechanical nuances driving their occurrence. A profound comprehension in this realm holds the promise of paving the way for innovative, patient-specific treatment modalities.

Historically, the understanding and categorization of ankle fractures have been shaped and refined by various classification systems and research breakthroughs. A quintessential example is the Lauge-Hansen's classification system. Conceived in 1950, this system offers a systematic approach to deciphering ankle fractures, and remarkably, it continues to retain its relevance in contemporary clinical practice [10]. Further enriching the orthopedic community's knowledge pool, ST Hansen and colleagues, in their seminal work in 2000, provided insights into the pathological underpinnings of ankle fractures. They pinpointed the concurrence of rotational and axial foot loading as a primary mechanism culminating in joint disruption [7]. This foundational knowledge was built upon by subsequent researchers. For instance, Weber et al.'s investigations in 2004 shed light on specific features and characteristics of posterior malleolus fractures [8]. Not to be outdone, Haraguchi et al., in 2006, furnished the medical fraternity with a nuanced classification delineating diverse fracture lines and patterns inherent to ankle fractures [9].

### The role of fracture fragments and the limitation of existing studies

In the nuanced landscape of orthopedic research, the implications of fracture fragments have long been a focal point, given their palpable impact on joint biomechanics. Both meticulous clinical observations and advanced model simulations underscore the significance of these fragments, especially in the context of heightened joint contact stress. This stress exacerbation is particularly concerning, considering its pivotal role in catalyzing the onset and progression of post-traumatic arthritis, a complication that can dramatically compromise joint function and patient quality of life [11, 12]. Inspired by these revelations, scholars like De Vries et al. and Bekerom et al. vociferously championed the merits of internal fixation, especially when the fractured fragment of the posterior ankle represents a substantial portion (more than 25%) of the tibial pilon [13, 14]. Yet, while their pioneering work has undeniably advanced our understanding, it is not devoid of limitations. A glaring oversight in their investigations pertains to the negligence of angle variations at the fracture site. Such variations could bear profound implications for treatment outcomes and prognosis. Addressing this lacuna forms a principal objective of the ongoing study.

### Emergence of finite element analysis (FEA) in stress analysis

To bridge the gap in our current understanding, researchers have sought to leverage the finite element analysis (FEA), a state-of-the-art numerical calculation technology built upon the principles of mechanics analysis, to examine the stress distribution in the ankle joint. By harnessing high-resolution computed tomography (CT) datasets, researchers can meticulously craft three-dimensional anatomical representations, offering unparalleled insights into the biomechanics underpinning posterior malleolus fractures.

The academic contributions in this domain are both rich and varied. For instance, the work of Guan et al. stands out for its deep dive into the influence of fracture lines on critical biomechanical parameters, such as stress distribution, contact topology, and the articular surface's relative displacements—attributes indispensable to joint stability [15]. Furthering this narrative, Evers et al. and Qiang et al. delved into the biomechanical repercussions of a posterior malleolar fragment (PMF) that constitutes less than a quarter of the joint's surface area. Their studies meticulously dissected the nuances of pressure gradients and stability, while also exploring the postoperative biomechanics of calcaneal fractures, especially concerning the strategic placement of the sustentaculum screw [16, 17]. Complementing these insights, Alonso-Rasgado et al.'s trailblazing work employed advanced 3-D computational modeling to delineate the multifaceted interplay between fragment dimensions, displacements, and the resultant biomechanical effects on ankle stability and contact dynamics [18].

### Study aim and justification

In the realm of orthopedic biomechanics, finite element analysis (FEA) has been an instrumental tool, unveiling intricate biomechanical insights that traditional methods might have overlooked. However, one salient observation is the conspicuous absence of attention given to the sagittal angle variations in the majority of FEA studies. Such oversights are no mere academic nuances; they have tangible clinical implications that could significantly influence therapeutic decisions and prognosis. Given this backdrop, the primary objective of our study is to bridge this knowledge chasm. We endeavor to craft a meticulous, high-fidelity ankle joint model, designed with precision to encapsulate and elucidate the minute details of stress distribution across the ankle's contact surface. This model is not merely a static representation; it is geared to be a dynamic tool, capable of simulating and analyzing the biomechanical consequences of various sagittal fracture angles on the posterior lateral malleolus fracture scenario.

Our hypothesis posits that sagittal angle variations, even those that might appear subtle, can have profound effects on contact surface pressures. We believe that understanding these variations and their subsequent biomechanical ramifications is pivotal. Not only does it deepen our academic understanding, but it also provides orthopedic surgeons with invaluable insights that could shape the trajectory of diagnosis, management, and therapeutic interventions for posterior malleolus fractures.

## Methods

### Data collection

Our data collection protocol began with the acquisition of 3D computed tomography (CT) scans from consenting, healthy volunteers. Special emphasis was placed on ensuring that the subjects maintained a neutral foot position throughout the scanning process, minimizing potential variations and artifacts. Spanning the length of the tibia to the depths of the foot, this rigorous scanning regimen produced a voluminous dataset: precisely 380 high-resolution CT images of the right foot, each rendered at a resolution of 1024 × 1024 pixels. For ease of data management and subsequent analyses, these images were diligently archived in the DICOM format.

The subsequent challenge was to morph these individual CT slices into a cohesive 3D representation, achieved using the capabilities of Mimics 16.0. This medical imaging software facilitated the seamless conversion of the 2D CT images into an integrated 3D geometric model of the right ankle. The end product of this stage was a comprehensive STL format geometric model that included not just the bones, but also accounted for the nuanced surface topographies of the lower tibia, fibula, talus, and calcaneus.

**Fig. 1:**
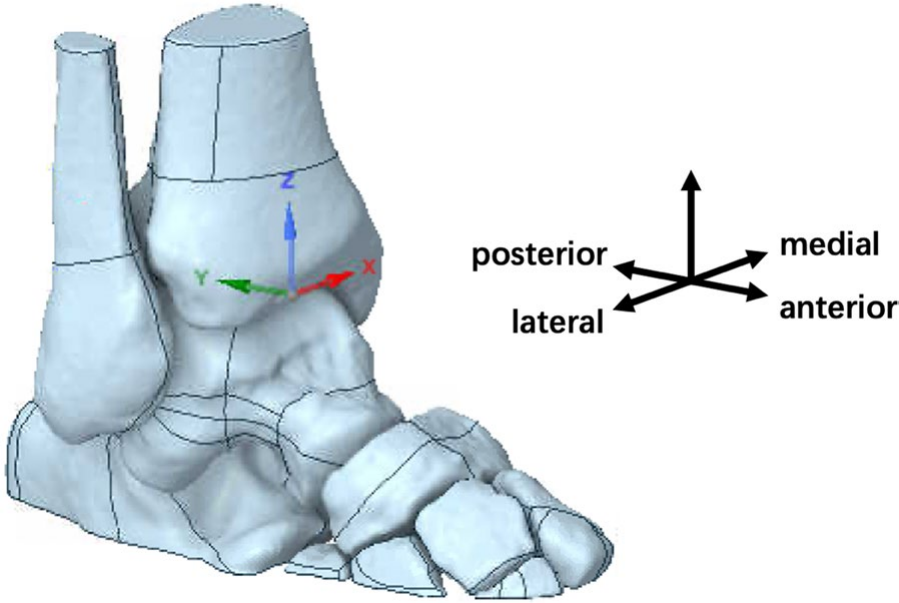
3D surface model of volunteer A's right ankle reconstructed from CT imaging

However, raw imaging data, even when reconstructed, can be marred by noise, irregularities, and imperfections. Therefore, to ensure the fidelity and accuracy of our model, we embarked on a refining process using SolidWorks 2021 [19]. Through a combination of surface smoothing algorithms, noise reduction techniques, and subdivision strategies, we sculpted a pristine, high-definition model of the ankle joint. This polished model, which serves as the bedrock for our subsequent analyses, is vividly depicted in Fig. 1. Additionally, the dimensional accuracy and anatomical precision of this model were cross-verified using independent measurements, ensuring its readiness for the subsequent biomechanical simulations and analyses.

### Finite element analysis (FEA) model creation

The translation from an anatomically precise 3D surface model to a biomechanically FEA model presents its own set of challenges. First, we tapped into the capabilities of Geomagic Studio 11 [20], which played a pivotal role in the transformation of our STL surface model into a comprehensive 3D volumetric representation. Relying on the intricacies of the STL file's faceted data, we constructed an STP format volumetric model, ensuring that every nook and crevice of the anatomical geometry was reproduced. Beyond the hard bone structures, the delicate cartilaginous components of the ankle joint play an essential role in its biomechanics [21]. Recognizing this, we captured the cartilage's nuanced anatomy, ensuring that its spatial relationships and morphological features mirrored their real-life counterparts.

Equipped with a holistic STP model, our next port of call was the FEA software, Ansys 2021 [22]. This platform offered the requisite tools to establish a comprehensive FEA engineering project, where we delineated the regions of the model and allocated materials based on their anatomical and biomechanical characteristics. Our material selections, influenced by both historical research and current advancements, are detailed in Table 1. Particularly, the bone was conceptualized as an isotropic, linearly elastic substrate. Such a decision was informed by a myriad of past studies that explored the mechanical properties of osseous tissues [23–26]. The cartilage, renowned for its low friction coefficient (hovering below 0.0025) [27], was considered to partake in virtually frictionless interactions with adjacent bony structures.

**Table 1:** Material property settings

	Density (g/cm^3^)	Elasic modulus (MPa)	Possion ratio
Posterior tibiobular	1.94e^−3^	18.44	0.49
Articular cartilage	1.94e^−3^	0.83	0.49

Within our FEA project, we mapped the intricate contact interfaces between the articular cartilage and the underlying bone. Bridging different skeletal components, we synthesized a comprehensive static analysis model, which represented the full biomechanical gamut of the ankle joint. Relying on Ansys’ robust automatic mesh generation algorithm, we were bestowed with an expansive 3D ankle mesh model. This mesh comprised a 11,725,276 nodes and 8,521,685 elements [28], each contributing to the model's fidelity.

### Finite element analysis (FEA) fracture models across varying sagittal angles

Our intent was to simulate, with the utmost fidelity, the anatomical and biomechanical peculiarities that pertain to ankle fractures across varying sagittal angles. The crux of our fracture modeling was influenced by a groundbreaking retrospective study that delved deep into the sagittal angle theory of ankle fractures. This study had analyzed and interpreted computed tomography (CT) scans from a significant pool of fracture patients, unraveling the intricacies of the three-dimensional fracture line dynamics [15].

With this theoretical framework at our disposal, our initial endeavor was to establish a standardized coordinate system that would serve as the reference for all subsequent fracture simulations. Our choice of the *Z*-axis, denoting the heel-to-knee trajectory, was intuitive, given its alignment with the long axis of the tibia. Similarly, the *Y*-axis, tracing the line from the toe to the heel, was an embodiment of the foot's anteroposterior orientation. The *X*-axis, consequently determined using the right-hand rule, rounded off our orthogonal coordinate system.

**Fig. 2:**
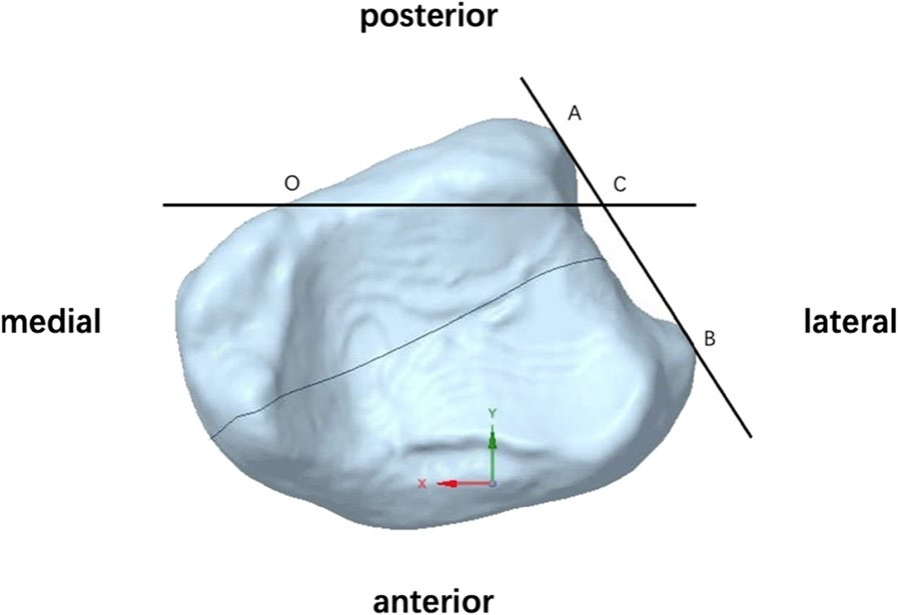
Bottom view of the fracture model, highlighting the fracture plane passing through OC

**Fig. 3:**
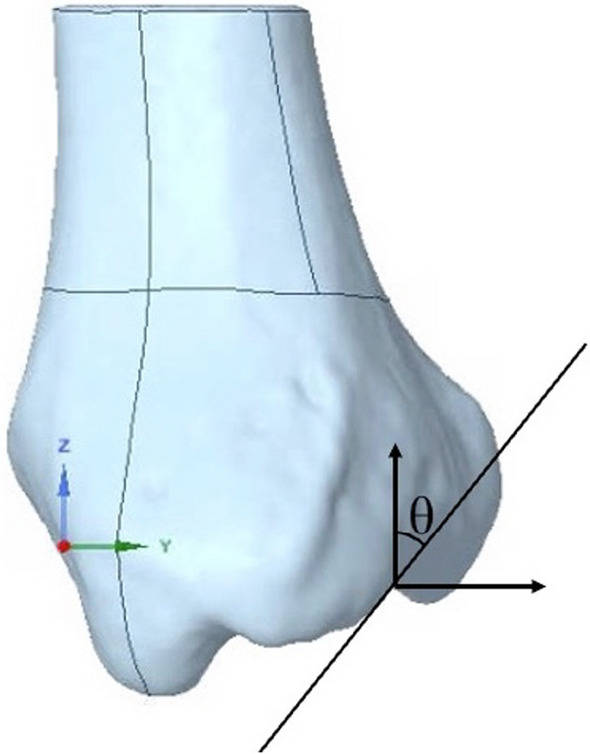
Side view of the fracture model, illustrating varying sagittal angles (*θ*) from 12° to 60°

For the sake of clarity and precision, we demarcated points A and B on the distal aspect of the tibia, where it met its tangent plane. Nestled comfortably on line AB was point C, whose location was predicated on capturing a quarter of AB's span. A line, OC, drawn parallel to the *X*-axis, emerged as a vital geometric entity (Fig. 2). This crafted geometry set the stage for defining the fracture plane. Visualized as the plane cleaving through OC and standing orthogonal to the *XY* plane, its alignment held the key to the fracture's sagittal angle dynamics. Consequently, the sagittal angle (*θ*) became emblematic of the tilt between our designated fracture plane and the *Z*-axis, providing a tangible metric to quantify the fracture's orientation (Fig. 3).

**Fig. 4:**
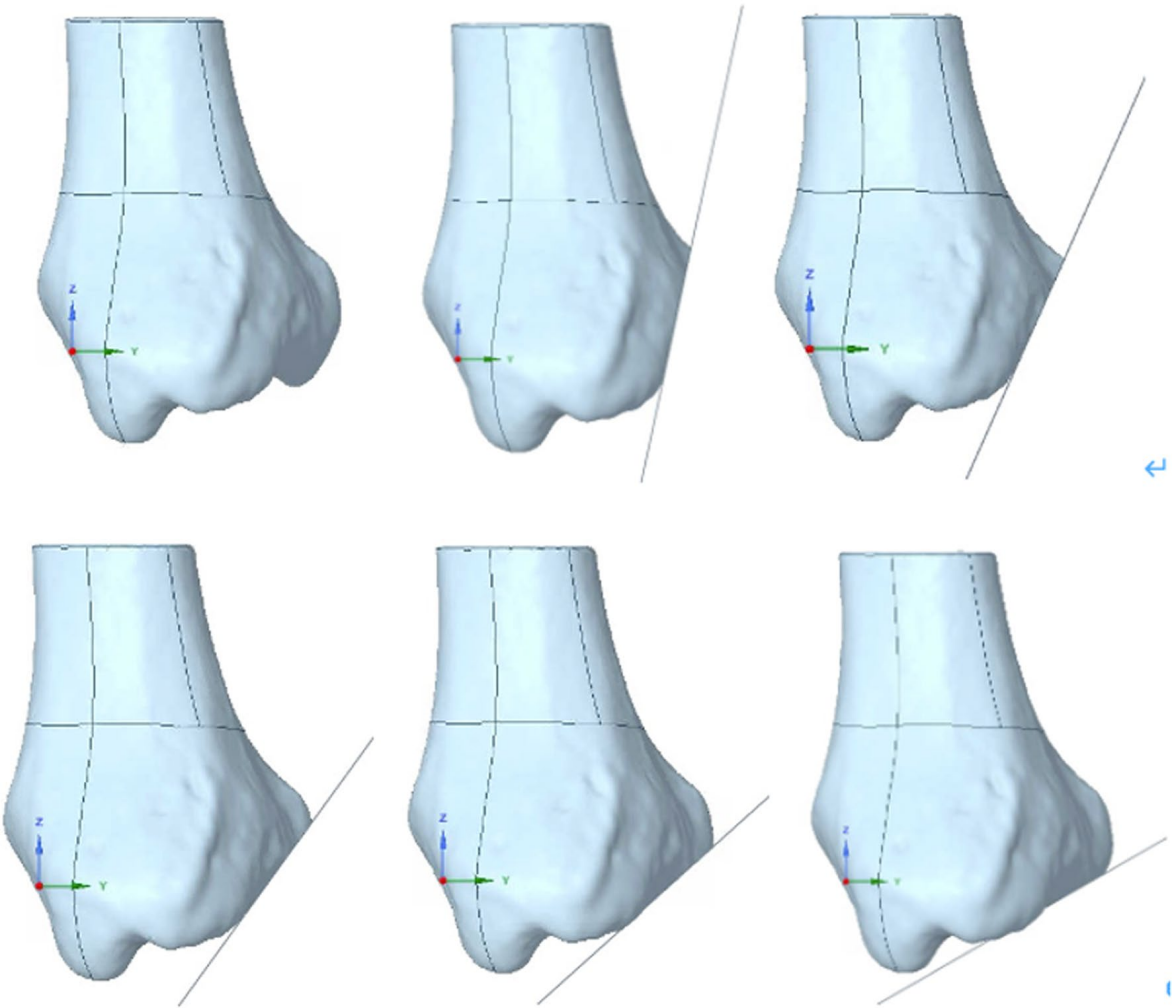
Comparison of the intact bone and five different fracture line locations. The top left image displays the intact bone, while the remaining images present varying sagittal angles from 12° to 60°

Then, we embarked on the construction of an ensemble of fracture models, each differing in its sagittal angle. Staggered at 12° intervals, this suite of models spanned angles from a modest 12° to an aggressive 60° (Fig. 4). Our choice of this specific range was motivated by its relevance to clinical scenarios and its potential to unveil nuanced biomechanical insights. Seamlessly integrating with Ansys Workbench, our modeling strategy culminated in the creation of five distinct FEA computation models.

### Boundary conditions

Our objective was to replicate the biomechanical nuances of bipedal standing—a complex interplay of skeletal alignment, muscular forces, and weight distribution. Historical precedence, enriched by extensive biomechanical research, has often employed an average human body weight of 70 kg as a standard proxy for load-bearing studies [29, 30]. Aligning with this established norm, we too anchored our simulations around this body weight, ensuring uniformity and comparability with prior investigations. In Ansys Workbench static model, we engineered a load distribution strategy. Recognizing the tibia's central role in weight-bearing during bipedal standing, we channeled 50% of the total gravitational force—translating to an axial load of 350N—directly onto its apex. This distribution ensured that the force vectors acted in line with the tibia's longitudinal axis, mirroring the physiological weight-bearing pattern. Meanwhile, to ground our models and provide stability, we rendered the basal surface of the calcaneus—often considered the pillar of the foot—inert and completely immobilized. This constraint mimicked the foot's natural stance on a firm surface, establishing equilibrium and preventing unwarranted translations or rotations.

The contact interface between the ankle bones is central to the joint's mobility and stress distribution. To capture their dynamic relationship, we instituted a contact condition that permitted only tangential displacements at the distal end of the ankle bone. This decision was informed by the biomechanical reality of the joint, where, despite considerable compressive forces, the articular surfaces glide over each other with fluidity.

## Results

In our endeavor to understand the biomechanics of the ankle joint, both in its pristine and fractured states, we commenced by investigating the joint surface contact area under a standard vertical load of 350N. The contact area in an unfractured model was determined to be 239 mm^2^. A retrospective analysis of this result allows for a gratifying comparison with prior cadaveric and FEA studies. To elucidate, Kimizuka et al. posited a mean contact area of 196.4 ± 64.4 mm^2^ [31], while Brown et al.'s investigations yielded a figure of 229 mm^2^ [32]. Further anchoring our findings in the realm of computational biomechanics, Alonso et al. detailed a contact area of 240 mm^2^ through their FEA approach [18].

Advancing to the realm of stress dynamics, the maximum contact stress on the joint surface was earmarked at 3.76 MPa. This quantification finds resonance with the research insights of Kimizuka et al., who recorded a stress of 4.4 MPa [31], and Guan et al., who documented a comparable stress of 3.79 MPa [15].

**Fig. 5:**
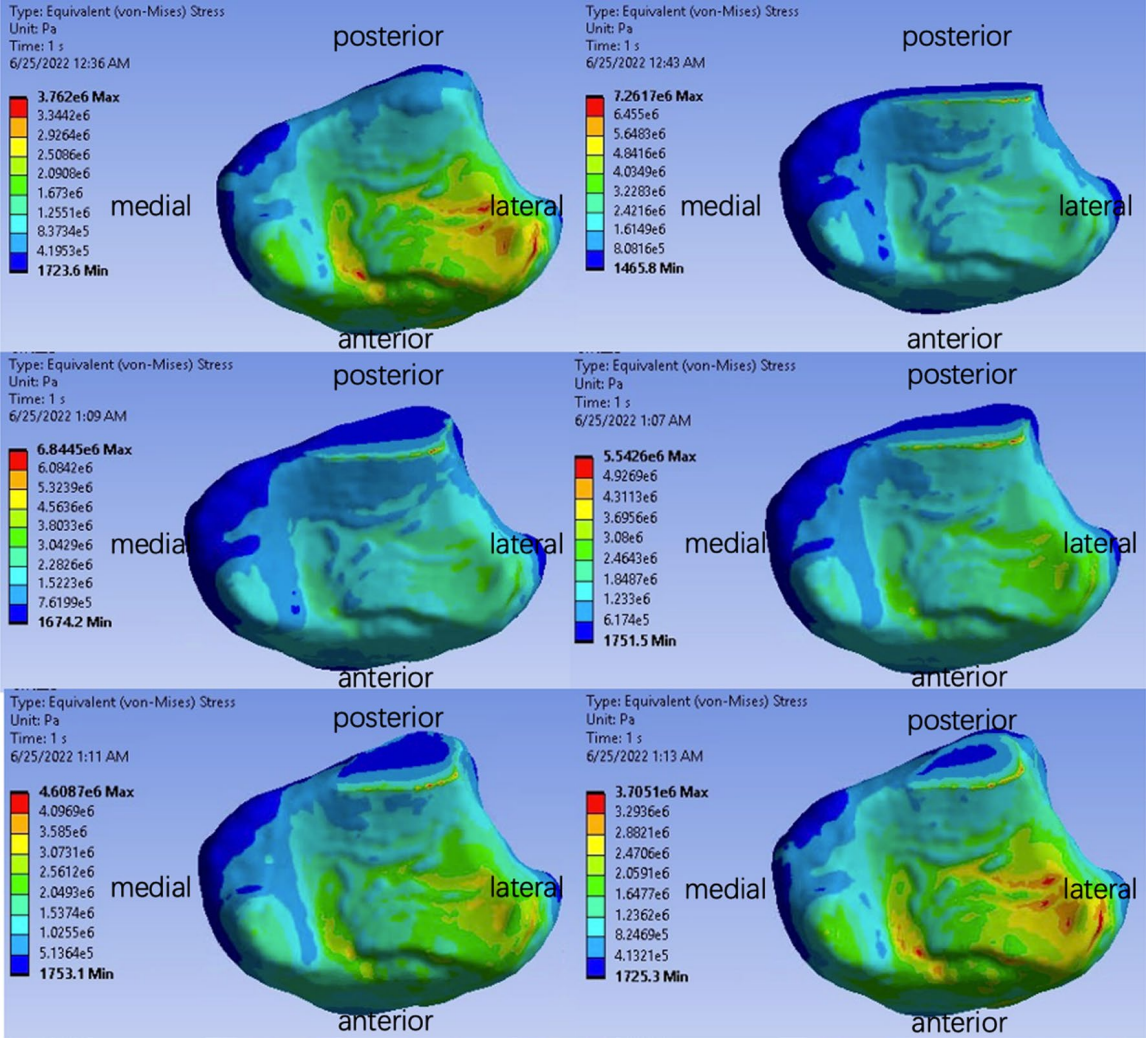
Stress distribution of the contact surface for different models

**Fig. 6:**
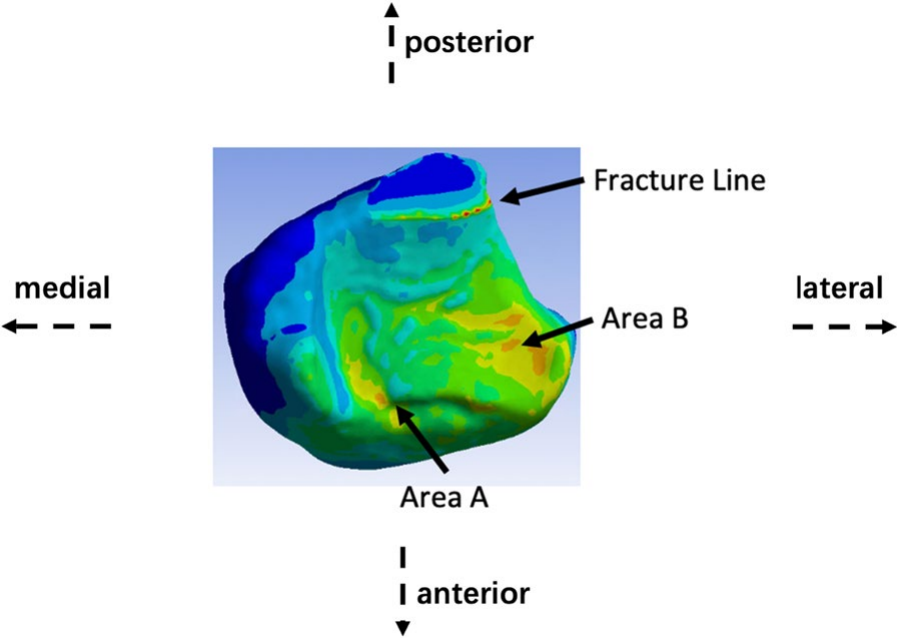
Areas of peak contact surface pressure

The introduction of fractures into our models unveiled a nuanced landscape of stress distribution, as vividly depicted in Fig. 5. A discerning observation was the emergence of contact surface pressure peaks delineated across three pivotal regions: A, B, and along the fracture trajectory. Providing anatomical context, Region A is situated in the anteromedial quadrant of the tibia, while Region B is localized to the depressed contour of the anterolateral tibial surface. A noteworthy adjunct here is Fig. 6—a bottom-up perspective, which casts the medial to the left of the posterior—this figure elucidates the spatial position of the fracture line, schematically simplified in alignment with the actual fracture trajectory observed in the volunteered subject.

**Fig. 7:**
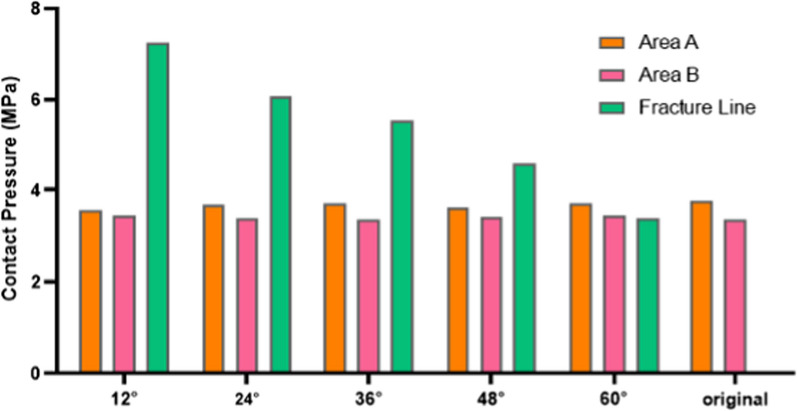
Contact pressure peaks at different fracture angles

The quintessence of our investigation, however, was captured in the granular analysis of localized pressure peaks, as chronicled across the six distinct scenarios presented in Fig. 5. Our synthesis of this data, encapsulated in Table 2 and Fig. 7, spotlighted a pivotal metric: the ratio of the fracture part's area to the holistic joint contact surface area, symbolized as *σ*. In our models, *σ* reached its zenith at 10.2% when the fracture was angled at 12°. Intriguingly, as the fracture angle augmented, *σ* embarked on a gradual descent. Analyzing regions A and B, the contact pressures sustained remarkable consistency, registering at 3.668 ± 0.118 MPa for Area A, and a slightly attenuated 3.396 ± 0.053 MPa for Area B. A pronounced spike in pressure was observed along the fracture line, climaxing at a formidable 7.261 MPa at the 12° angle—a value surpassing the peaks of both regions A and B, and tapering off with amplified fracture angles. Our results found symmetry with the groundbreaking work of Fitzpatrick et al., where compressive ankle experiments highlighted peak pressures oscillating between 7 and 9 MPa [33]. We have also calculated the average contact pressure on the joint surface at varying sagittal angles. The average contact pressure demonstrates a slight decrease as the sagittal angle increases from 12° to 60°, from 2.287 to 2.188 MPa. This trend offers further insight into the distribution of stress across the contact surface and complements our findings on peak stress concentrations.

**Table 2:** Contact pressure peaks at different fracture angles

Fracture angle	12°	24°	36°	48°	60°	Original
Surface ratio *σ*	10.2%	8.6%	7.5%	6.8%	6.2%	0%
Area A contact pressure (MPa)	3.550	3.672	3.699	3.620	3.705	3.762
Area B contact pressure (MPa)	3.449	3.377	3.345	3.411	3.439	3.355
Fracture line contact pressure (MPa)	7.261	6.084	5.542	4.608	3.398	-
Average contact pressure on the contact surface (MPa)	2.287	2.265	2.248	2.225	2.219	2.188

## Discussion

Finite element analysis was employed in this study to provide insights into the stress distribution across the contact surface of the ankle joint, both in its natural state and under varied sagittal fracture line angles. Historically, posterior malleolus fractures have been associated with ankle instability. This is because the ligament pull that follows such fractures can lead to movement of the fracture fragment and consequently disrupt the weight-bearing surface's integrity [34, 35]. Given the intrinsic difficulties associated with obtaining precise laboratory-based spatial pressure measurements of the ankle [14, 40], this research offers a contribution.

The alignment of our model's stress predictions with those reported in empirical studies underscores its potential utility in a clinical setting. The maximum contact stress identified is particularly significant given its proximity to the values reported in cadaveric studies, which are often considered the gold standard for biomechanical analysis. This suggests that the model can reliably approximate in vivo conditions, providing a valuable tool for presurgical planning and postoperative evaluation. However, it is critical to note that while our model's contact stress predictions are within the range of those found in the literature, the exact values may vary due to differences in methodology, sample size, and the biomechanical properties assigned to the tissues in different studies. Despite these potential variations, the consistency of our results with those of Kimizuka et al. [31] and Guan et al. [15] reinforces the robustness of our modeling approach.

Through meticulous modeling, this study has brought to light three distinct stress concentration zones on the ankle's contact surface, namely the anterolateral tibia, the anteromedial tibia, and the fracture line itself. Notably, any fractures can potentially shift the apex of this stress triad, paving the way for possible ankle instability. While past studies have often generalized the stress distribution, our findings underscore the nuanced alterations that fractures introduce to the stress landscape [36]. Moreover, these stress hotspots also align with frequently observed clinical fracture sites, shedding light on the interplay between stress redistribution and the biomechanical consequences of fractures.

One of the standout revelations of this investigation is that the most significant stress is exerted at the fracture line when a fracture occurs. Delving into the data presented in Fig. 7, it becomes evident that larger sagittal angles (relating to smaller fracture fragments) see the stress points at the contact surface's three vertices exhibit only slight variations. In contrast, as the sagittal angle diminishes, stresses at both the posterolateral and posteromedial tibia largely remain static, but there is a notable spike in stress at the fracture line. Such stress escalation can compromise ankle stability. Past research endeavors have pointed out that an increase in joint contact stress, caused by fracture fragments, is a crucial factor influencing the onset of post-traumatic arthritis [37, 38]. The study presented here suggests that sagittal angles below 60° might pose considerable risks to ankle stability. Thus, careful evaluation of the sagittal fracture angle can be pivotal for informed therapeutic decision-making. Prolonged stress exertion on the soft tissues at the fracture site might also hasten the onset of traumatic arthritis. Earlier works have identified a robust link between extensive ankle fracture fragments and the emergence of traumatic arthritis, emphasizing the need for internal fixation in cases where the fractured articular surface area of the posterior malleolus exceeds 25% [13, 14, 39]. The present study reinforces these viewpoints.

The inclusion of average contact pressure data in our analysis provides a more nuanced understanding of the stress distribution across the contact surface of the ankle joint. As observed, the average contact pressure slightly diminishes with larger sagittal angles. This observation suggests that while peak stress concentrations are critical for assessing the risk of acute damage at specific points, the average stress distribution also has implications for the overall biomechanical integrity of the joint. Specifically, the reduction in average contact pressure at larger angles may reflect a distribution of force that could mitigate the risk of concentrated stress leading to fracture propagation or joint degeneration.

Heralding the merits of FEA, this research showcases its potential as an indispensable tool for a deeper understanding of posterior malleolus fractures. It provides clinicians with granular data about stress distribution alterations across various sagittal angles, thus enriching the decision-making process, especially concerning treatment interventions. The inferences drawn from this study hint that surgical procedures might be essential for fractures with reduced sagittal angles to prevent joint instability and the possible complications that may ensue.

However, it is essential to acknowledge the study's limitations. In our finite element model, we chose to reconstruct the bones without separation to preserve the overall structural integrity for our initial simulations. This decision was informed by a focus on global stress distribution patterns that might influence clinical decision-making in the context of ankle stability and the risk of arthritis development postfracture. While our model provides a robust representation of the joint's biomechanical behavior under a standardized load, we acknowledge that it does not account for the potential separation of bone fragments that can occur in situ. This limitation notwithstanding, the model offers valuable insights into the initial biomechanical environment postfracture. However, we recognize that the behavior of individual bone fragments under load, and their contribution to localized stress alterations, represents a critical aspect of postfracture biomechanics. Future studies incorporating separated bone fragments within the model could illuminate the detailed effects of fragment size, location, and mobility on joint biomechanics. Furthermore, assumptions of linear elasticity and isotropy for the bones were made, which might not entirely mirror real-world situations where bones exhibit nonlinear elastic and anisotropic traits. Moreover, the study's scope was restricted to simulating a static stance. Future research endeavors could encompass walking simulations and juxtapose findings with clinical evidence for a more comprehensive picture. The authenticity of the current model awaits experimental verification, underscoring the need for more extensive studies before the model's findings can be fully integrated into clinical applications.

## Conclusion

The present study, utilizing finite element analysis (FEA), has illuminated the intricacies of stress distribution across the ankle joint's contact surface, both in its natural state and when subjected to different sagittal angles of fracture lines. Our findings accentuate the pivotal role of the contact surface in ensuring ankle stability and the subsequent alterations that fractures can introduce to its stress profile. This research has identified three specific zones of stress concentration, with fractures having the potential to shift the peak stress, which may lead to ankle instability. Furthermore, the study highlights the significance of sagittal angles in determining the stability of the ankle post-fracture. A sagittal angle below 60° is identified as a potential risk factor for compromised ankle stability. This provides a crucial reference point for clinicians, assisting in the decision-making process for therapeutic interventions.

## Data Availability

The datasets used and analyzed during the current study are available from the corresponding author on reasonable request.
